# Physical Activity of Secondary School Adolescents at Risk of Depressive Symptoms

**DOI:** 10.1111/josh.12911

**Published:** 2020-06-17

**Authors:** Karel Frömel, Lukáš Jakubec, Dorota Groffik, František ChmelÍk, Zbyněk Svozil, Michal Šafář

**Affiliations:** ^1^ Faculty of Physical Culture, Palacký University Olomouc třída Míru 117, Olomouc 771 11 Czech Republic; ^2^ Institute of Sport Science The Jerzy Kukuczka Academy of Physical Education in Katowice Mikolowska 72a, Katowice 40‐065 Poland

**Keywords:** recommendations, IPAQ, pedometer, quality of life, well‐being

## Abstract

**BACKGROUND:**

The aim of the study is to analyze the associations between depressive symptoms (DS), well‐being and different types of physical activity (PA) in adolescents.

**METHODS:**

Overall, 368 girls and 228 boys aged 15‐19 years were involved in the research. To explore the composition of weekly PA, we used the IPAQ‐Long questionnaire, and a pedometer was used to monitor weekly PA. The prevalence of DS was diagnosed by the Bern Subjective Well‐Being Questionnaire and the WHO‐5 Well‐Being Index.

**RESULTS:**

The girls and boys who reported the most DS and the lowest level of well‐being had significantly less weekly recreational PA. The girls who reported the fewest DS had a 2.12 times greater odds of meeting the 11,000 steps/day recommendation than did the girls with the most DS, whereas we did not detect statistically significant differences in rates of meeting the recommendation in the boys with distinct levels of DS.

**CONCLUSIONS:**

The study confirms the stronger negative associations between DS and PA, especially among girls. The greatest opportunities for behavioral change in adolescents at the highest risk of DS are in the promotion of recreational PA.

Physical activity (PA) is associated with improved cognitive functioning and life‐long mental health enhancement; however, it is challenging to quantify its benefits in relation to mental health.^1^ It is clear that PA has the potential to protect adolescents against depressive symptoms (DS), but evidence‐based findings on the topic are limited.^2^ The focus on the associations between DS, well‐being, and PA in adolescents is a current research issue, especially because adolescents manifest a significant decline in PA with age,^3^, ^4^, ^5^ as well as an increase in rates of overweight and obesity,^6^ an increase in their level of sedentary behavior,^7^ and a sleep deficit.^8^ We can observe the increase in DS relating to the transition to secondary school, especially at age 15‐16,^9^, ^10^ as well as the associations between obesogenic risks and DS in adolescents.^11^

The decline in PA is associated with an increase in sedentary behavior, which increases the risk of anxiety.^12^ Sedentary behavior and physical inactivity are associated with anxiety and DS.^13^ Strong consistent evidence was found for the relationship between both DS and leisure screen time, especially when that screen time accounts for more than 2 or 3 hours per day.^14^ Strong associations between screen‐time leisure sedentary activities, obesogenic risks, and depression in adolescents were also shown by Hoare et al.^11^ Furthermore, the level of adolescents' mental stress increases at school,^15^ and educational stress at school is not adequately compensated for by regeneration activities, in particular, by adequate PA.^16^, ^17^

It is difficult to quantify the benefits of PA that are associated with improving the quality of life, including the reduction of DS.^1^ Interventions that are focused on the promotion of PA and the improvement of well‐being and thus protection against DS do not always yield the assumed results.^18^ The effects of specific intervention programs on decreasing the prevalence of DS have not yet been sufficiently convincing.^19^ Therefore, it is necessary to extend the body of research on the associations between DS, well‐being, and the overall PA level by analyzing different types of PA and the varying frequency, volume, and intensity of the PA. The importance of research on the associations between DS and various types of PA in adolescents, both in terms of PA volume and intensity, is highlighted by Kleppang et al.^20^ From this perspective, the most significant contribution was made by a large cross‐sectional study in the United States comprising 1.2 million people; however, the participants were over 18 years of age.^21^ The study's finding that exercising for more than 6 hours per week can be associated with deteriorated mental health needs to be respected at the level of secondary schools as well. This result is similar to the finding that the effects of higher PA intensity on the reduction of DS in adolescents' depression treatment are unclear.^22^ Nevertheless, there is a growing body of evidence supporting the claim that PA may be protective against depression, regardless of age and geographical region.^23^

Most studies address the associations between PA and DS at the level of registered patients with clinical symptoms.^24^, ^25^ Therefore, health prevention studies need to be conducted under usual school conditions as well. For instance, the results of research studying the influence of college students' participation in school sports on their well‐being and activity levels confirmed the indirect influence, particularly through their body image, instrumentality, and physical competence. Hence, the authors consider the promotion of girls' involvement in school sports to be very important for their well‐being at secondary schools.^26^ Intervention based on the Sports‐based Youth Development mentorship program, which was carried out at 12 secondary schools in Hong Kong, showed that the sensible use of curricula and support by schools and municipalities can be helpful in addressing the issue of growing mental stress and the rise in the prevalence of adolescents' mental disorders.^27^ The integration into curricula of the Creating Opportunities for Personal Empowerment programme,^28^ which is focused on physical and mental well‐being, yields similarly promising results. A study conducted at German schools^29^ shows that physically active adolescents suffer from fewer psychosocial problems than do their less active counterparts.

Sufficient evidence confirming the positive associations between higher levels of PA and a lower occurrence of DS is lacking among post‐secondary students, as shown by Dogra et al.^30^ We can state that this lack applies to the level of secondary schools as well. Our research question is, thus, as follows: In which types of PA can we find the greatest differences between the adolescents who are at the highest and lowest risk of DS and what are the differences between these 2 groups in rates of meeting the PA recommendations?

Therefore, the aim of the study is to analyze the associations among DS, well‐being, and different types of PA in adolescents, under usual school conditions, and to propose measures to improve the physical behavior of the secondary school youth who are at the highest risk of DS.

## METHODS

### Participants

We conducted our research in 16 secondary schools of the Czech Republic between 2015 and 2016. The schools were selected based on the long‐term cooperation of universities and secondary schools in particular regions, and we also took different types of secondary schools and varying sizes of cities into account (<30,000; 30,000‐100,000; >100,000 inhabitants). A total of 94% of students and their parents gave us their consent to participate in the study. Overall, 368 girls and 228 boys aged 15‐19 years participated in our research (Table 1). From the original set of 729 participants, 133 participants were excluded from the research for failing to comply with the eligibility criteria.

**Table 1:** Sample Characteristics

		Age (years)		Weight (kg)		Height (cm)		BMI (kg/m^2^)		Steps/day	
Characteristics	N	M	SD	M	SD	M	SD	M	SD	M	SD
Boys	228	16.72	1.04	70.12	11.48	179.52	7.37	21.73	3.15	10,419	3378
Girls	368	16.61	0.96	59.35	8.94	167.77	6.51	21.06	2.77	10,540	2821

BMI, body mass index; M, mean; SD, standard deviation.

### Instruments and Procedure

The same research team organized and carried out the study at all schools. The initial training in PA monitoring was given and the instructions for completing the questionnaires were delivered to the students in their computer classrooms. All of the students were registered in the web application called the “International Database for Research and Educational Support” (INDARES; www.indares.com). After completing the questionnaires in Indares, the students received training in using pedometers to monitor weekly PA and in recording the monitoring data to record sheets and to the Indares application.

#### *Retrospective assessment of PA, well‐being, and DS*

We used the “International Physical Activity Questionnaire—Long Form” (IPAQ‐LF)^31^ to identify the students' composition of weekly PA in the last 7 days. The long version of the questionnaire was chosen to obtain more detailed information about their PA structure over a week, especially at school, during transportation, at home and around the house, in leisure time, vigorous PA, moderate PA, and walking. The Czech version of the IPAQ‐LF questionnaire was prepared following the translation guidelines.^32^ Pearson's correlation coefficient, as an indicator of concurrent validity between weekly PA (METs‐min) in the IPAQ‐LF questionnaire and weekly step count (steps/week) equals to r = .283. The internal consistency reliability coefficient Cronbach's alpha is α = 0.845. The Czech version of the IPAQ‐LF questionnaire has already been applied in previous studies.^17^, ^33^, ^34^ Among others, the questionnaire includes items querying the sorts of PA that are most commonly performed during the year.

We processed the data in line with the IPAQ manual, but we multiplied the MET‐min for vigorous physical activity (VPA) by a coefficient of 6 METs. The total daily time of PA reported must not have exceeded 600 minutes, and the total MET‐min per week must not have been over 20,000 MET‐min. The reason for these modifications was to not disturb the proportional composition of the weekly PA. The most widely recognized PA recommendation of 60 minutes of PA/day^35^, ^36^ was changed to at least 60 minutes of PA (any type of PA available in the questionnaire) for 5 or more days a week and, concurrently, at least 20 minutes of VPA 3 or more times per week.^37^ The recommendations had to be met in at least one type of PA, which is a challenging criterion.

We used the Bern Questionnaire on Subjective Well‐being^38^ in the modified and standardized Czechoslovak version.^39^ In this study, we analyzed only the responses to the DS‐related questions. We split the adolescents into quartiles, (Q1‐Q4), with girls and boys being separate.

The index of emotional well‐being was investigated using the WHO (Five) Well‐Being Index (1998 version)‐Czech (https://www.psykiatri‐regionh.dk/who‐5/Pages/default.aspx). The index has been used widely over the world for long time and is an appropriate measure to be used in research studies to compare well‐being between groups of respondents.^40^ As in the case of DS, the adolescents were divided into 4 groups (Q1‐Q4). A score of below 13 points, which identifies the adolescents as being at the highest risk of DS, corresponded to the Q4 quartile. This result indicates poor well‐being and is an indication for testing for depression under ICD‐10.

#### *Weekly monitoring of PA by pedometers*

We used the Yamax Digiwalker SW‐700 Pedometer (Yamax Corporation, Tokyo, Japan) to objectively monitor the weekly PA. To avoid the reactivity of PA monitoring.^41^ the adolescents wore the pedometers immediately after the initial training session on the first day. The actual weekly monitoring was launched on the following day and lasted for a week. The adolescents wore the pedometers for the entire day (except for swimming or bathing), from morning (after personal hygiene) until going to bed (evening hygiene). Six students who had swimming training as a part of their daily programs were excluded from the sample. We used 11,000 steps/day as a daily PA recommendation for both boys and girls, which does not contradict similar recommendations.^42^

### Data Analysis

In Statistica version 13 (StatSoft, Prague, Czech Republic) and SPSS version 22 (IBM SPSS, Inc., Chicago, IL) programs, we used descriptive statistics to provide basic characteristics of the sample, cross‐tabulations to assess differences in rates of meeting the PA recommendation among various groups, Kruskal‐Wallis ANOVA to analyze associations between weekly PA and different levels of, repeated measures ANOVA to evaluate weekly PA monitored by pedometers, binary logistic regression with enter method to estimate odds of meeting the PA recommendation, and ŋ^2^, ŋ_p_^2^, and r effect size coefficients. The effect size coefficients were interpreted as follows: 0.01‐0.059, small effect size; 0.06‐0.139, medium effect size; and ≥0.14, large effect size. The level of statistical significance was set at p < .05. The differences in PA that were greater than or equal to 2000 steps/day, and 10% of weekly PA recommendations were considered to be of practical significance.

## RESULTS

### The Associations Between Different Levels of DS and Types of PA According to the IPAQ‐LF Questionnaire

Girls (Q4) with most DS had significantly less recreational PA than did girls (Q1) with the least DS (Mdn ± IQR = 693 ± 2007 METs‐min versus 1314 ± 1630 METs‐min). The same pattern was also observed for VPA (Mdn ± IQR = 0 ± 1440 METs‐min versus 1080 ± 1560 METs‐min) (Table 2). The boys (Q4) with the highest level of DS had significantly less recreational PA than did boys (Q1) with the least DS (Mdn *±* IQR = 1080 ± 2061 METs‐min versus 2026 ± 2982 METs‐min).

**Table 2:** The Association Between Adolescents' Weekly Physical Activity (METs‐min/week) and Depressive Symptoms

		Depressive Symptoms										
Lowest (Q1)		Lower (Q2)		Higher (Q3)		Highest (Q4)	
Physical Activity	Sex	Mdn	IQR	Mdn	IQR	Mdn	IQR	Mdn	IQR	H	p	η^2^
School	Boys	720	2021	1170	2157	1107	2006	760	2255	0.67	.881	0.001
Girls	1223	2277	743	1403	614	2058	571	2145	3.09	.378	0.005
Transportation	Boys	912	1482	594	1089	776	1320	693	1458	3.17	.367	0.005
	Girls	873	1155	809	1527	749	1331	990	1584	2.29	.514	0.004
Home	Boys	555	1088	540	1350	255	995	540	1255	3.71	.294	0.006
	Girls	300	830	400	898	360	655	420	920	0.38	.944	0.001
Recreation	Boys	**2026**	**2982**	1200	2081	1129	1866	**1080**	**2061**	**12.95**	**.005**	**0.022** ^*^
	Girls	**1314**	**1630**	1155	1863	773	1242	**693**	**2007**	**8.53**	**.036**	**0.014** ^*^
Vigorous	Boys	1560	3240	1080	1920	750	2010	1080	2160	4.66	.199	0.008
	Girls	**1080**	**1560**	540	1620	615	1440	**0**	**1440**	**11.88**	**.009**	**0.020** ^*^
Moderate	Boys	1305	2968	1520	2795	1060	2160	1155	2603	2.19	.533	0.004
	Girls	1130	1130	1000	1850	860	1220	808	1645	1.78	.619	0.003
Walk	Boys	1536	3218	1254	2244	1304	2393	1469	1733	1.80	.614	0.003
	Girls	1716	2657	1584	2277	1287	2970	1724	2855	1.35	.717	0.002
Total	Boys	5448	7593	4710	6015	3999	3753	4471	4490	3.59	.309	0.006
	Girls	4556	4165	3600	4518	3810	4586	4028	5057	2.63	.453	0.004

Note: bold font indicates statistical significance *p* < .05.

H, Kruskal‐Wallis test; IQR, interquartile ranges; Mdn, median values; η^2^, Cohen's effect size; p, significance level; η^2^, *0.01 ≤ η^2^ < 0.06 small effect size.

Compared with 17.6% of the adolescents with the highest level DS, 29.4% of the adolescents with the lowest level of DS met the recommendation of at least 60 minutes of PA for 5 or more days a week and, at the same time, at least 20 minutes of VPA for 3 or more days a week (χ^2^ = 5.14, p = .023, r = .137). Team sports were ranked first among the most common types of PA for both boys and girls, totaling 39.0% among the study adolescents. Half of those adolescents reported the lowest level of DS, while in the group of adolescents with other types of PA, 35.8% had the lowest level of DS (χ^2^ *=* 5.05, p = .025, r = .137).

### The Association Between Varying Well‐Being Indices and PA Types According to the IPAQ‐LF Questionnaire

The associations between the WHO (5) Well‐Being Index and PA types correspond to the abovementioned results. The girls who had the lowest well‐being index (ie, the Q1 group of adolescents with the most DS) performed significantly less recreational PA than did the girls with the highest well‐being index (ie, the Q4 group of adolescents with the fewest DS) (H = 11.10, p = .011, η^2^ = 0.019^*^) (Mdn ± IQR = 630 ± 1737 METs‐min versus 1208 ± 1845 METs‐min), VPA (H = 8.83, p = .030, η^2^ = 0.015^*^) (Mdn ± IQR = 270 ± 1260 METs‐min versus 780 ± 1680 METs‐min), moderate PA (H = 9.35, p = .025, η^2^ = 0.016^*^) (Mdn ± IQR = 775 ± 1115 METs‐min versus 1280 ± 1903 METs‐min) and overall PA (H = 11.15, p = .011, η^2^ = 0.019^*^) (Mdn ± IQR = 3123 ± 4290 METs‐min versus 5016 ± 5208 METs‐min). Compared with the boys who had the highest well‐being index, those boys with the lowest well‐being index had significantly less PA only in the dimension of recreational PA (H = 9.11, p *=* .028*,* η^2^ = 0.015^*^) (Mdn ± IQR = 885 ± 2145 METs‐min versus 1807 ± 2237 METs‐min).

Compared with 15.6% of adolescents who had the lowest well‐being, 26.1% of adolescents with the highest well‐being met the recommendation of at least 60 minutes of PA for 5 or more days a week and, at the same time, at least 20 minutes of VPA for 3 or more days a week (χ^2^ = 5.02, p = .025, r = .128). Forty‐seven percent of the respondents who participated in team sports reported a statistically significant higher well‐being and therefore a lower threat of DS compared with 35.1% of the adolescents in other types of PA (χ^2^ = 3.99, p = .046, r = .099).

### The Association Between Different Levels of Depressive Symptoms and Weekly PA by Steps/Day

**Figure 1:**
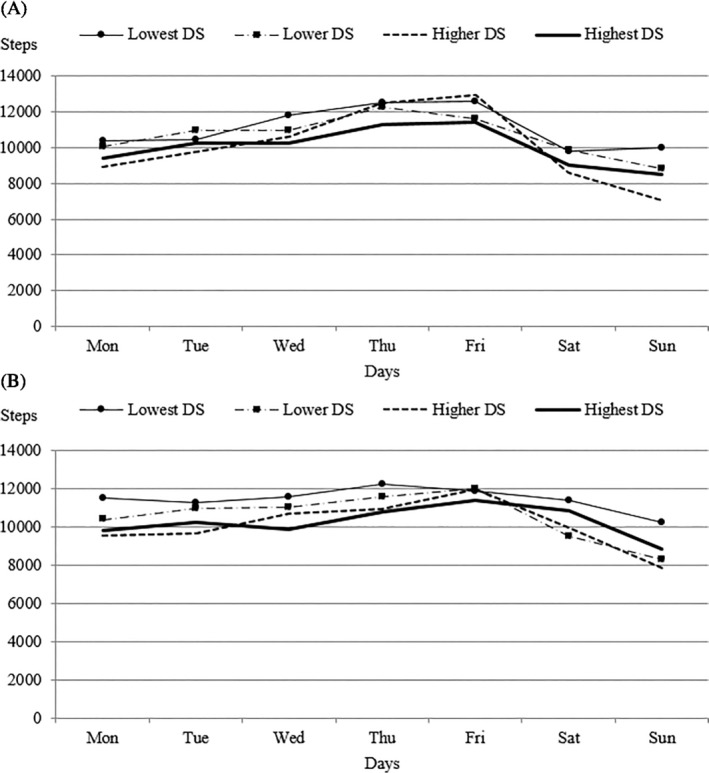
Mean Step Count/Day for Each Day of the Week Split for Boys (A) and Girls (B) with Different Levels of Depressive Symptoms (DS)

The differences between the adolescents with the most and fewest DS by day of the week were not statistically significant either in girls (F_(18,1344)_ = 0.943, p = .523, η_p_^2^ = 0.012) or in boys (F_(18, 1344)_ = 1.57, p = .058, η_p_^2^ = 0.013) (Figure 1). Overall, regardless of sex, the group with most DS (10,163 steps/day on average) reached both statistically and practically significant fewer steps/day than did the group with fewest DS (12,319 steps/day on average), (F_(3,588)_ = 3.76, p = .011, η_p_^2^ = 0.019).

### The Association Between Different Well‐Being Indices and Weekly PA by Steps/Day

**Figure 2:**
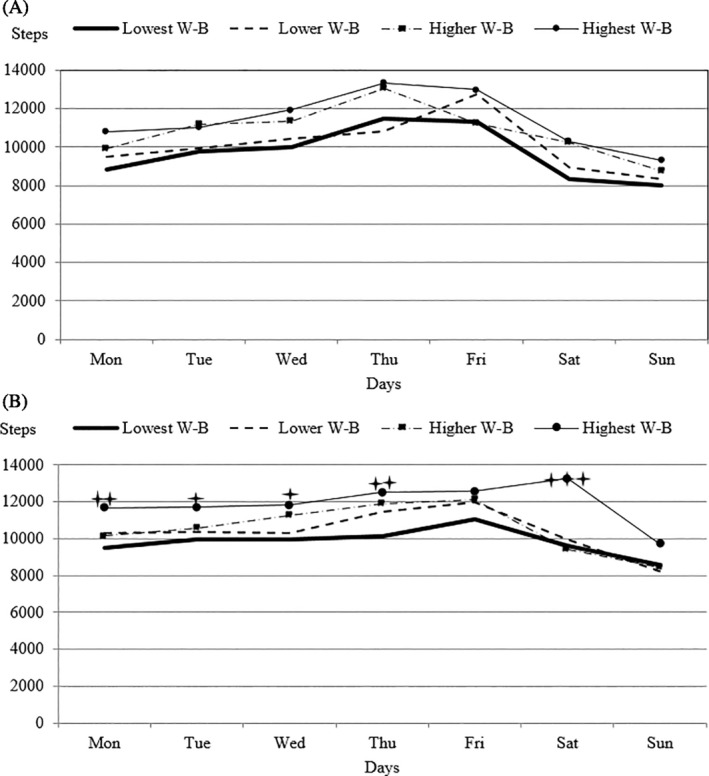
Mean Step Count/Day for Each Day of the Week Split for Boys (A) and Girls (B) with Different Levels of Well‐Being (W‐B)

The girls with the lowest well‐being index also had significantly fewer daily steps than did the girls with the highest well‐being index (F_(18,2184_) = 1.74, p = .027, η_p_^2^ = 0.014) on Monday (p = .008), Tuesday (p = .046), Wednesday (p = .032), Thursday (p = .004), and Saturday (p < .001) (Figure 2). Using the repeated ANOVA, we did not observe significant differences by particular days of the week in boys (F_(18,1344)_ = 0.767, p = .741, η_p_^2^ = 0.010). Overall, regardless of sex, the group with the lowest well‐being index reached 8779 steps/day, while those adolescents who reported the highest well‐being reached 11,683 steps/day (F_(3,588_) = 9.72, p < .001, η_p_^2^ = 0.047).

### The Association Between Different Levels of DS, Well‐Being, and PA on School and Weekend Days

On school days, the girls with the most DS reached an average of 10,277 steps/day (boys—10,656 steps/day), and the girls with the fewest DS averaged 12,393 steps/day (boys—11,515 steps/day), which we consider to be a practically significant difference in girls. The boys with the lowest well‐being index averaged 10,356 steps/day (girls—10,441 steps/day) and the boys with the highest well‐being index averaged 11,686 steps/day (girls—11,921 steps/day).

On weekend days, the boys with the most DS reached an average of 8645 steps/day (girls—10,207 steps/day) and those with the fewest DS reached an average of 9191 steps/day (girls—12,494). The boys with the lowest well‐being index reached an average of 8147 steps/day (girls—9997 steps/day), and those with the highest well‐being index reached an average of 9617 steps/day (girls 12,560 steps/day) on weekends. We find the differences in the mean step counts on the weekends between the Q1 and Q4 groups of groups to be practically significant both for DS and well‐being.

### Rates of Meeting the 11,000 Steps/Day Recommendation by Depressive Symptoms and Well‐Being

**Figure 3:**
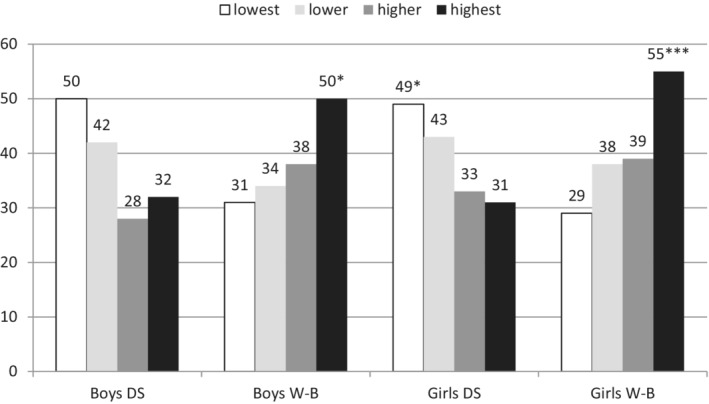
Rates of Meeting the 11,000 Steps/Day Recommendations for Boys and Girls with Different Levels of Depressive Symptoms (DS) and Well‐Being (W‐B)

The recommendation of 11,000 steps/day was met by 32.3% of the boys with the most DS, compared with 50.0% of the boys with the fewest DS (χ^2^ = 3.26, p = .071, r = .169). However, the difference between them was not statistically significant (Figure 3). Nonetheless, we consider the results to be practically significant. More pronounced and statistically significant differences were found in the girls (χ^2^ = 5.70, p = .017, r = .175). A total of 30.9% of the girls with the most DS met the steps/day recommendation, compared with 49.3% of the girls with the fewest DS. The recommendation of 11,000 steps/day was met by a statistically significant lower number of boys in the lowest well‐being group (31.3%) than of boys in the highest well‐being group (50.0%) (χ^2^ = 4.18, p = .041, r = .185). The pattern was similar in girls, with 29.4% of girls in the lowest well‐being group meeting the steps/day recommendation versus 55.1% of the girls in the highest well‐being group meeting the recommendation (χ^2^ = 12.11, p < .001, r = .253).

The girls reporting the fewest DS had 2.12 times greater odds of meeting the 11,000 steps/day recommendation than did the girls reporting the most DS (OR = 2.12, CI = 1.14‐4.14, p = .018), whereas we did not find statistically significant differences in the rates of meeting this recommendation in boys with distinct levels of DS.

Similarly, the girls with the highest level of well‐being had 2.80 times greater odds of meeting the 11,000 steps/day than did the girls with the lowest well‐being (ie, the most DS) (OR = 2.80, CI = 1.50‐5.2, p = .001). Additionally, the boys with the highest well‐being had 2.19 times greater odds of meeting the 11,000 steps/day than did the boys with the lowest well‐being (ie, the most DS) (OR = 2.19, CI = 1.50–4.67, p = .042).

## DISCUSSION

The major finding of this study is that the greatest disproportion of PA in the boys and girls who reported the most DS and the lowest well‐being, compared to those who reported the least DS and the highest well‐being, was observed in the dimension of recreational PA. This result is in spite of the fact that we can expect significant health and mental benefits of recreational PA in conjunction with school PA, both in elementary schools^43^ and in secondary schools.^44^ Compared with the other types of PA in the IPAQ, the domain of recreational PA provides a higher chance of participation in the preferred and most popular types of PA, more freedom to decide about participation in organized or unorganized PA, and more freedom in determining PA volume or intensity. Therefore, the diagnostics of PA preferences stand out, especially in the context of the possibility of complying with these preferences in the form of recreational PA in adolescents' leisure time.^37^

Associations between DS, PA types and the ways in which young people participate in PA are scarcely researched. Nevertheless, some of the findings are beneficial for improving mental health in secondary schools. For instance, stronger associations between lower levels of PA and an increase in DS were found among Icelandic adolescents aged 15‐16 years; furthermore, the authors observed a greater impact on reducing DS by participation in organized sports in girls than in boys.^9^ Adolescents' participation in school sports can be protective against poor mental health in early adulthood and may act as a significant predictor of DS.^45^ Thus, the monitoring and promotion of extracurricular sports as a part of the Comprehensive School Physical Activity Program (CSPAP),^46^, ^47^ as well as socio‐emotional prevention/intervention programs,^48^ is very timely and necessary. PA performed in sport clubs is also related to a lower likelihood of DS.^20^

The finding that the adolescents who were mostly involved in team sports showed fewer DS than did adolescents who were involved in other sports is in line with the results of a large‐scale study that was conducted in the United States, according to which team sports are strongly associated with improvements in mental health.^21^ Similarly, Arat and Wong^49^ observed a positive effect of team sports, particularly on loneliness and anxiety, which are related to DS. PA through team sports supports protection against DS in 12‐ to 13‐year‐old adolescents.^50^ Nonetheless, a change in PA climate, which is focused on mastery and learning instead of competition and performance, as emphasized by Holt et al.^51^ is necessary in terms of well‐being and DS. School‐based applied physical activities should promote good school climate. This is because the positive school climate, also enhances odds for improvement of adolescents' depression literacy.^52^

The finding in our research that a quarter of the adolescents reported DS corresponds to the findings of Khan and Burton,^53^ who also found a higher incidence of DS in 25% adolescents; however, this finding was in a group of adolescents who showed a high amount of leisure‐time screen time (ie, more than 2 hours per day). Because more leisure time spent in front of the screen is associated with more DS in adolescents, the psychological impact of the substitution of this time for physical activity and improved dietary behaviors^14^ should be examined. Blough and Loprinzi^54^ provide evidence that a short‐term weekly decrease in habitual PA causes a rise in DS in young active adults. However, it is still unclear whether these negative effects are a consequence of an increase in sedentary behavior or a decrease in moderate‐to‐vigorous physical activity (MVPA).

The fact that a recommendation of at least 60 minutes of PA for 5 or more days a week and, concurrently, at least 20 minutes of VPA for 3 or more days a week (according to the results from the IPAQ‐LF questionnaire) is met only by a low percentage of adolescents, especially those with the most DS, is in accordance with the results that we have already found in Central European conditions. These recommendations are met by 21.3% of boys and 12.6% of girls.^36^ According to numerous studies, most adolescents do not achieve the recommendation of 60 minutes of PA a day.^55^ Currie et al.^56^ reported that 77‐85% of European adolescents do not meet the MVPA recommendation of 60 minutes of daily PA. Similarly, the findings of the Centers for Disease Control and Prevention^57^ show that 73.9% of secondary school students do not meet the daily MVPA recommendation, and only 24% boys and 9% girls do so in Australia.^58^ Also in schools in Spain, Grao‐Cruces et al.^59^ found that PA levels during school hours, recess, and PE lessons in children and adolescents are very low.

Another serious finding of our research is that neither boys nor girls with the most DS and the lowest well‐being compensate for this emotional condition by engaging in PA in their after‐school time or on the weekends. In addition, studies have shown that the students with the highest mental load from their lessons do not compensate for this stress by engaging in PA during school recesses^15^ or on weekend days.^17^ This result is a challenge for teachers, physical education teachers, school managers, parents, and all institutions that create conditions for leisure‐time PA.

### Limitations

We consider the failure to carry out a randomized nationwide selection of schools and classes, mainly due to the excessive administrative activities that disrupt the educational process at schools, as a limitation. Furthermore, for the abovementioned reasons, as well as the difficulty of organizing the research and of preserving normal conditions, it was not appropriate to repeat the questionnaire survey investigating DS, well‐being, and the subjective estimate of PA for the monitoring week, which is a limiting factor. The simplified DS characteristics also do not allow us to distinguish between emotional, cognitive, and somatic levels of these symptoms, which should be addressed in future research.

## Conclusions

The finding that a quarter of the adolescents reported DS is a challenge to make changes in the school lifestyle, eliminate unnecessary DS and promote well‐being, even in a demanding educational program. The promotion of recreational PA among youth at the highest risk of DS during the after‐school time of the CSPAP calls for special attention. Moreover, it is necessary to consider the specific features of girls' PA with regard to their leisure‐time PA. To promote the rates of youths meeting the PA recommendations, which could lead to decline in incidence of DS among those adolescents who are at the highest risk in terms of their mental health, school programs should pay special attention to Mondays, and we should also support PA on the weekend days.

### IMPLICATIONS FOR SCHOOL HEALTH

To promote adolescents' mental health, we propose to promote extracurricular PA programs in the CSPAP for adolescents with DS more efficiently, with a strengthened focus on girls, who not only perceive and understand emotions better than boys but they also perceive higher amounts of stress at an older age.^47^ Moreover, we suggest to promote recommendations for school PA in specific segments of the school day more efficiently,^15^ to recognize Monday as a critical day regarding PA in school education programs, to focus on improving the quality of teaching and to utilize the effects of team sports to promote adolescents' mental health.

We consider the following measures to be suitable for promotion of adolescents' school health:

- To increase interest of school administration in use of simple diagnostics of adolescents' mental health in the association with the PA recommendations in specific segments of a school day (WHO‐5 Well‐Being Index and simple PA recommendations, expressed in step count, appear to be a feasible alternative).
- To support compensation for adolescents' educational stress by continuous development of physical and depression literacy in PE and also other school subjects.
- To promote efficiency of CSPAP by coordination with school socio‐emotional prevention program.
- To support especially team‐oriented PA in all forms of school physical activities (recesses, classroom physical activity breaks, PE lessons, extracurricular physical activity). The PA of this sort induces positive school climate and is more appealing to the adolescents with lower levels of PA and higher mental risks.
- To eliminate the risk of the weekend days and Monday, which display the lowest levels of PA in adolescents at risk of DS, by the offer of individual physical activity programs with the use adequate wearables.

### Human Subjects Approval Statement

All procedures were reviewed and approved by the chair of the Ethics Committee of Human Research of the Faculty of Physical Culture, Palacký University Olomouc (decision no. 24/2012). Parental consent to participate in the study was obtained for all adolescents in the sample.

### Conflict of Interest

All authors declare no conflicts of interest.
